# Key data elements for a successful pediatric rheumatology virtual visit: a survey within the PR-COIN network

**DOI:** 10.3389/fped.2024.1457607

**Published:** 2024-10-22

**Authors:** Y. Ingrid Goh, Meghan E. Ryan, Shoghik Akoghlanian, Rajdeep Pooni, Julia G. Harris, Danielle R. Bullock, Sheetal S. Vora, Tzielan C. Lee, Shirley M.L. Tse, Fatima Barbar-Smiley

**Affiliations:** ^1^ Division of Rheumatology, The Hospital for Sick Children, Toronto, ON, Canada; ^2^ Child Health Evaluative Sciences, SickKids Research Institute, Toronto, ON, Canada; ^3^ Division of Rheumatology, Allergy & Immunology, Department of Pediatrics, University of Minneapolis, Minneapolis, MN, United States; ^4^ Department of Rheumatology, Nationwide Children’s Hospital, Columbus, OH, United States; ^5^ Division of Pediatric Rheumatology, Department of Pediatrics, Stanford Medicine Children’s Health, Palo Alto, CA, United States; ^6^ Stanford School of Medicine, Stanford University, Palo Alto, CA, United States; ^7^ Division of Rheumatology, Department of Pediatrics, Children’s Mercy Kansas City, Kansas City, Missouri, United States; ^8^ School of Medicine, University of Missouri-Kansas City, Kansas City, MO, United States; ^9^ University of Minnesota Medical School, University of Minnesota, Minneapolis, MN, United States; ^10^ Department of Pediatric Rheumatology, M Health Fairview Masonic Children’s Hospital, Minneapolis, MN, United States; ^11^ Department of Pediatrics, Atrium Health Levine Children’s, Charlotte, NC, United States; ^12^ Wake Forest School of Medicine, Wake Forest University, Charlotte, NC, United States; ^13^ Department of Pediatrics, Faculty of Medicine, University of Toronto, Toronto, ON, Canada

**Keywords:** pediatric rheumatology, telemedicine, virtual, data documentation, eHealth, telehealth, telerheumatology, quality of care

## Abstract

**Introduction:**

Juvenile idiopathic arthritis (JIA) is the most common childhood rheumatic disease which is commonly monitored by a combination of history, physical examination, bloodwork, and imaging. The COVID-19 pandemic prompted a rapid shift to telemedicine to ensure that patients continued to receive healthcare. The shift to telemedicine changed the methodology and ability of healthcare providers to monitor their patients' progress, as they were unable to perform direct hands-on assessments. The following survey sought to understand the impact of switching pediatric rheumatology healthcare delivery from in-person to telemedicine modality. Specifically, it sought to examine the rate of collection of critical data elements (CDE) for monitoring JIA disease activity and outcomes, barriers and facilitators to its collection, opinions on difficulty and importance of collecting CDE over telemedicine, tools and electronic medical record modifications that facilitated CDE collection, and other data elements that were important to collect during telemedicine visits.

**Methods:**

A cross-sectional survey was sent to healthcare providers at all PR-COIN centers who saw patients using telemedicine. Qualitative data was analyzed using descriptive statistics and qualitative data was analyzed using an inductive approach.

**Results:**

Survey respondents reported that they documented the CDE at least 75% of the time. Barriers to assessing and documenting critical data elements included (1) the inability to palpate or visualize all joints over telemedicine, (2) connectivity issues, and (3) forgetfulness with collecting all CDE. Respondents suggested using reminders within the electronic medical record to prompt documentation completeness and improve reliability. They also suggested including medication adherence, quality of life, and patient/caregiver satisfaction with their telemedicine experience as part of their documentation. A few centers reported that they had established processes to assist with data collection in advance of the telemedicine visit; however, the variation in responses reflects the need to standardize the process of providing care over telemedicine.

**Discussion:**

Multiple barriers and facilitators to collecting CDE during telemedicine visits exist. Given that a proportion of the population will continue to be seen over telemedicine, teams need to adapt their practices to consistently provide high-quality care over virtual platforms, ensuring that patients at any institution receive a standardized level of service.

## Introduction

Juvenile idiopathic arthritis (JIA) is a rare, childhood chronic condition which is estimated to affect between 2 and 8 million children worldwide (1, 2). Although JIA can be effectively managed with advanced anti-rheumatic therapies, ineffective treatment can result in pain, disability, and potential vision loss from uveitis (3, 4). Healthcare providers document various indicators to monitor JIA disease activity (5–10). These may include active joint count and provider global assessment (PGA) of disease activity. Patient reported indicators are also documented including pain scores and patient global assessment (PtGA) (11). Ultimately, reliable collection of these metrics influence disease monitoring and management, thereby impacting patients' long-term outcomes.

The Pediatric Rheumatology Care and Outcomes Improvement Network (PR-COIN) learning network, currently comprised of 23 medical centers and parents across the United States and Canada, works collaboratively to identify and close gaps in healthcare for patients with JIA (12). They employ a “treat-to-target” strategy based on outcomes reported by both healthcare providers and patients or families is used to optimize care (13). PR-COIN previously established a set of quality measures to improve the care of children with JIA (10). Twenty measures including 10 outcome measures, 5 process measures, 4 data measures, and 1 balancing measure were included (10). Of the 20 measures identified, six were designated critical data elements (CDE): morning stiffness, joint pain, number of active joints, uveitis screening, PtGA, PGA of disease activity, which were deemed important for monitoring JIA disease activity and outcomes (10, 13). Consistent documentation and tracking of these CDE have enabled healthcare providers at PR-COIN sites to monitor their patient outcomes (10, 13). Monitoring of CDE has enabled healthcare providers to improve the outcomes of patients with JIA (14).

Access to care is essential for careful monitoring and timely management of JIA. Access to pediatric rheumatology care has long been a challenge due to the limited workforce in this field (15–17). The COVID-19 pandemic and its calls for physical distancing and quarantine further exacerbated the already limited access to healthcare providers and services (18, 19). During the pandemic, telemedicine use rose and became an alternative or complementary visit type to traditional in-person visits (20, 21). Coordinated design, evaluation, testing, adaptation, and sharing of best practices across rheumatology clinics is essential to optimize the care provided to patients with JIA in telemedicine settings (22–24).

Evidence supporting the provision of care using telemedicine in rheumatology in both the adult and pediatric populations has existed prior to the COVID-19 pandemic, but its adoption increased out of necessity for continued provision of care during the COVID crisis (25–30). An initial survey estimated that three-quarters of PR-COIN sites did not utilize telemedicine prior to the pandemic but were subsequently able to implement telemedicine by March 2020 (18). Providers felt that about half of their population could be safely and effectively seen over telemedicine (18). Although these centers were able to adapt to providing healthcare over telemedicine in the short-term, providers expressed concerns about the long-term effects of utilizing virtual care (18). This finding was not surprising given the hands-on examination is central to the examination process of pediatric rheumatology. During the pandemic, many PR-COIN site providers adopted the use of the Pediatric Gait, Arms, Legs, and Spine (PGALS) exam as an alternative to the hands-on exam (18, 31).

Recognizing the challenges of performing active joint count assessments over telemedicine, we wondered whether the shift of healthcare delivery to a virtual setting affected healthcare providers' ability to reliably collect all six CDE (10). We therefore sought to understand the healthcare providers' perspectives on the completion rates, barriers and facilitators to collecting CDE over telemedicine, which are important to successfully monitoring JIA disease activity and outcomes. The ultimate goal was to use these findings to design interventions to reduce these barriers, in turn, enabling more reliable collection of CDE via telemedicine, thereby improving the quality of healthcare provided over telemedicine to patients with JIA over telemedicine, resulting in better long-term outcomes.

## Materials and methods

A cross-sectional electronic survey was created by the PR-COIN Digital-Health workgroup to characterize healthcare providers' experiences with the collection and documentation of CDE during telemedicine visits. The survey asked respondents to indicate which CDE they collected during telemedicine visits; their comfort level of collecting CDE over telemedicine; barriers to collecting CDE over telemedicine; tools that facilitated CDE collection; indicate which of the six CDE was most important to capture; which CDE was most difficult to capture, and what other data elements they thought was worth capturing during telemedicine visits. These responses were based on respondents' active recall and not an actual audit. Finally, respondents were asked to share changes which they instituted or had planned for their site's electronic medical record system as a result of delivering care over telemedicine.

A link to the voluntary, anonymous survey was sent to the lead principal investigators (PIs) of the 21 PR-COIN centers (number of existing center at the time of the study). The PIs were requested to share the survey link to their center's clinical staff who saw patients with JIA using telemedicine. The PIs were asked to confirm the number of recipients who they had sent the survey to in order to determine the denominator. This strategy was employed to avoid sending the survey to an outdated member mailing list. Participants provided implied consent to participate in the survey. The survey, which was conducted from August-September 2020.

The survey data was collected and managed using Research Electronic Data Capture (REDCap) (32, 33). REDCap is a workflow methodology and software solution designed for rapid development and deployment of electronic data capture tools to support clinical and translational research (32, 33).

Quantitative results were analyzed using descriptive statistics, and qualitative results were thematically analyzed using an inductive approach.

The PR-COIN registry and network-related collaborative quality improvement activities, including member surveys that are used as part of continuing quality improvement, were approved by Cincinnati Children's Medical Health Center's Institutional Review Board (IRB).

## Results

### Survey distribution and response rate

The survey was sent to the lead contact at 21 PR-COIN sites in the United States and Canada. Nineteen of 21 PR-COIN sites were represented in the survey response. Some sites were solely comprised of pediatric rheumatologists, while other sites were composed of a multidisciplinary team which included fellows and practitioners (medical professionals who are not physicians but have received additional training and are qualified to perform many similar functions as a physician, such as prescribing medications, diagnosing, treating, and managing patient care). Teams ranged from two staff pediatric rheumatologists at the smallest site to 14 staff pediatric rheumatologists, fellows, and practitioners. Fourteen (73.7%) sites reported having more than five pediatric rheumatologists at their sites.

The survey was sent to a total of 121 clinical staff who saw patients with JIA using telemedicine. A total of 119 (98.3%) completed the survey. Of the responses received, 103/119 (86.6%) surveys were fully completed, while 16 were partially completed.

Eighty-two (68.9%) respondents indicated they were staff pediatric rheumatologists, 24 (20.2%) were fellows, and the remainder (10.9%) were practitioners.

### Collection and level of comfort collecting critical data elements during telemedicine visits

Respondents indicated that the six CDE data elements were collected more than half of the time (Table 1). The most documented CDE over telemedicine was morning stiffness 104/119 (87.4%), while the least commonly documented was arthritis-related pain score 77/119 (64.7%) (Table 1). Only one (0.8%) respondent indicated that they did not collect any of the 6 CDE identified by PR-COIN.

**Table 1:** Collection of critical data elements by respondents.

Critical data element	Count	(%)
Morning stiffness	104/119	(87.4%)
Completion of uveitis screening as per recommendations	98/119	(82.4%)
Provider global assessment	90/119	(75.6%)
Active joint count	88/119	(73.9%)
Patient global assessment	78/119	(65.5%)
Arthritis-related pain score	77/119	(64.7%)
Do not collect any critical data elements	1/119	(0.8%)

**Figure 1:**
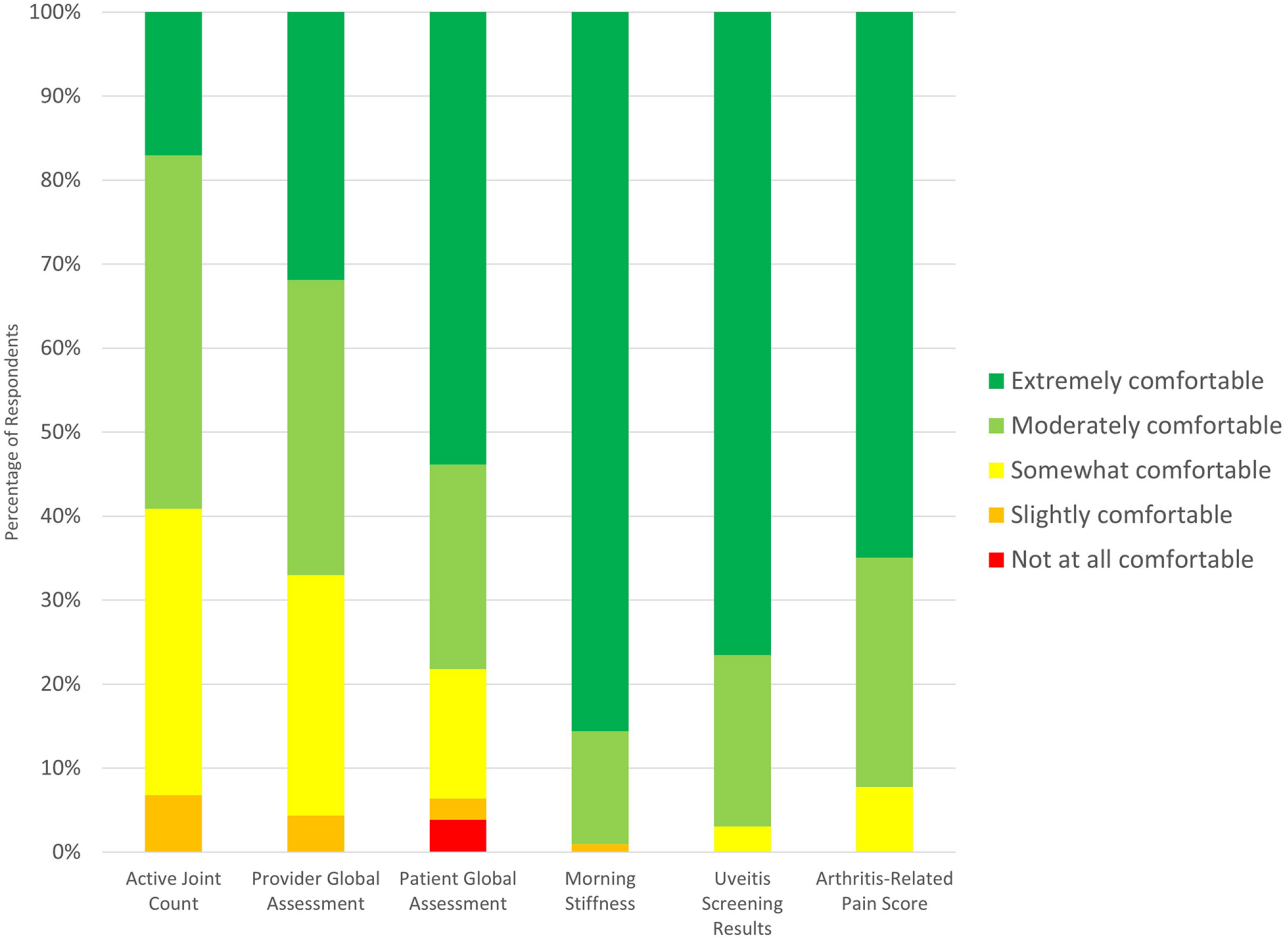
Comfort collecting critical data elements over telemedicine.

Of the 104 individuals indicating that they documented morning stiffness during telemedicine visits, 51 (49.0%) respondents indicated that they documented this parameter at every visit (Table 2). 89/104 (85.6%) respondents indicated that they were extremely comfortable documenting morning stiffness during telemedicine visits (Figure 1).

**Table 2:** Percentage of time critical data element collected by respondents.

	Active joint count	Provider global assessment	Patient global assessment	Morning stiffness	Uveitis screening results	Arthritis-related pain score
Never	0	0	0	0	0	0
Rarely, in less than 10% of the chances when I could have	0	2 (2.2%)	1 (1.2%)	1 (0.9%)	0	3 (3.9%)
Occasionally, in about 30% of the chances when I could have	3 (3.4%)	5 (5.6%)	5 (6.4%)	3 (2.9%)	0	1 (1.3%)
Sometimes, in about 50% of the chances when I could have	6 (6.8%)	8 (8.9%)	10 (12.8%)	2 (1.9%)	3 (3.1%)	10 (13.0%)
Frequently, in about 70% of the chances when I could have	22 (25.0%)	18 (20.0%)	18 (23.1%)	6 (5.8%)	21 (21.4%)	13 (16.9%)
Usually, in about 90% of the chances I could have	31 (35.2%)	32 (35.6%)	25 (32.1%)	41 (39.4%)	41 (41.8%)	28 (36.4%)
Every time	26 (29.5%)	25 (27.8%)	14 (17.9%)	51 (49.0%)	33 (33.7%)	22 (28.6%)

Of the 98 individuals indicating that they documented uveitis screening during telemedicine visits, 33 (33.7%) respondents indicated that they documented this parameter at every visit (Table 2). 75/98 (76.5%) respondents indicated that they were extremely comfortable documenting uveitis screening during telemedicine visits (Figure 1).

Of the 90 individuals indicating that they documented PGA during telemedicine visits, 25 (27.8%) respondents indicated that they documented this parameter at every visit (Table 2). 27/89 (30.3%) respondents indicated that they were extremely comfortable documenting PGA during telemedicine visits (Figure 1).

Of the 88 individuals indicating that they documented active joint count during telemedicine visits, 26 (29.5%) respondents indicated that they documented this parameter at every visit (Table 2). 15/88 (17.0%) respondents indicated that they were extremely comfortable documenting active joint count during telemedicine visits (Figure 1).

Of the 78 individuals indicating that they documented PtGA during telemedicine visits, 14 (17.9%) respondents indicated that they documented this parameter at every visit (Table 2). 42/78 (53.8%) respondents indicated that they were extremely comfortable documenting PtGA during telemedicine visits (Figure 1).

Of the 77 individuals indicating that they documented arthritis-related pain scores during telemedicine visits, 22 (28.6%) respondents indicated that they documented this parameter at every visit (Table 2). 50/77 (64.9%) respondents indicated that they were extremely comfortable documenting arthritis-related pain scores during telemedicine visits (Figure 1).

Overall, respondents appeared to be more comfortable collecting data which were reported by patients than data based on their assessment over telemedicine.

### Barriers to collection of critical data elements

Forgetfulness and not knowing which tool to use to collect data were barriers for the collection of all CDE. Barriers to collecting uveitis screening results included that patients did not have their last screening date readily available during their visit nor did they have their results. Other barriers to not collecting PGA and active joint CDE included the inability to see and palpate joints and being too distracted with technical issues of using telemedicine. Additional barriers for active joints collection included difficulty assessing small joints or detecting subtle swelling, difficulty assessing young patients, and being too distracted with technical issues. PtGA collection barriers included not having the proper resources to facilitate its collection over telemedicine and an element not typically collected by a specific site. Other barriers to collecting arthritis-related pain scores included lack of proper resources to facilitate its collection over telemedicine and patients lack clarity in knowing whether their pain was related to arthritis.

### Tools used to assist with collection of critical data elements

When asked what tools clinicians were using to collect CDE, the respondents from 16/19 (84.2%) centers indicated that they were using the pGALS to support the evaluation of joints. 9/19 (47.4%) centers reported that they had developed or had an existing mechanism to collect patient reported outcomes prior to the telemedicine clinic visit. 13/19 (68.4%) sites reported that they had existing reminders (e.g., forms/templates/flowsheets) or had created reminders in their electronic medical record system to remind them to collect CDE.

### Ranking of critical data elements by importance and assessment difficulty

When respondents were asked to rank which CDE they thought was most important of the six, the majority indicated that was the active joint count 74/103 (71.8%) (Table 3).

**Table 3:** Rank order of importance of critical data elements.

	Active joint count	Provider global assessment	Patient global assessment	Morning stiffness	Uveitis screening results	Arthritis-related pain
1	74 (71.8%)	16 (15.5%)	6 (5.8%)	4 (3.8%)	1 (1.0%)	2 (1.9%)
2	14 (13.6%)	31 (30.1%)	10 (9.7%)	25 (24.0%)	8 (7.7%)	16 (15.2%)
3	8 (7.8%)	21 (20.4%)	24 (23.3%)	21 (20.2%)	19 (18.3%)	10 (9.5%)
4	2 (1.9%)	14 (13.6%)	22 (21.4%)	24 (23.1%)	24 (23.1%)	18 (17.1%)
5	2 (1.9%)	15 (14.6%)	21 (20.4%)	19 (18.3%)	17 (16.3%)	31 (29.5%)
6	3 (2.9%)	6 (5.8%)	20 (19.4%)	11 (10.6%)	35 (33.7%)	28 (26.7%)

1 = Most important, 6 = Least important.

When respondents were asked to select which was the most difficult of CDE to collect during the telemedicine visit, the majority 79/109 (72.5%) indicated that it was the active joint count (Table 4).

**Table 4:** Select the most difficult critical data element to collect over telemedicine.

Active joint count	79 (75.2%)
Patient global assessment	12 (11.4%)
Provider global assessment	5 (4.8%)
Arthritis-related pain	5 (4.8%)
Uveitis screening results	4 (3.8%)
Morning stiffness	0

### Other elements to collect during telemedicine visits

When survey respondents were invited to suggest additional elements worth collecting during telemedicine visits, the majority suggested collecting a satisfaction survey regarding patient's telemedicine experience. Other suggestions included medication adherence, mood assessment, limitations in activities of daily living, quality of life, and the number of non-billable encounters that occurred over telemedicine.

### Modifications to electronic medical record system to delineate telemedicine visits

The majority [60/109 (55.0%)] of respondents indicated that their site had made changes to their electronic medical record system to indicate that visits were conducted over telemedicine. 22/109 (20.2%) respondents indicated that their electronic medical record system already had the capability of distinguishing which visits were conducted in-person and which visits were conducted over telemedicine. Eight (7.3%) respondents indicated that their site intended to make changes to their electronic medical record system in the future to enable them to distinguish which visits occurred in-person vs. over telemedicine.

## Discussion

The COVID-19 pandemic has resulted in significant changes in healthcare delivery in both the inpatient and ambulatory settings (34–36). For pediatric rheumatologists, this change has been most apparent in the outpatient setting given that many patients with chronic disease, including JIA, require frequent outpatient follow-up visits. Although the availability of telemedicine increases access for our patients (26, 37), we must consider not only access and acceptability, but also the quality of healthcare delivered over this medium, which may ultimately affect safety and patient outcomes (38). Our initial work (18) indicated that there was a significant variability in the reliable collection of many data elements needed for clinical care at a PR-COIN site level. This study focused on individual provider practices. We observed that the majority of providers were collecting CDE at least 60% of the time when seeing patients over telemedicine. Certain CDE were collected more reliably than others. This may have been related to similarities in how the CDE is administered during in person visits. For example, morning stiffness is often verbally asked of the patient or proxy during their in-person clinic visits.

The inability to perform hands-on physical examinations mostly affected provider's ability to determine active joint count and, in turn, the PGA. This uncertainty, in turn, made them less comfortable in documenting their findings into the patient's electronic medical record.

Morning stiffness and uveitis screening were the most commonly collected CDE. However, when providers were asked to rank the importance of these elements, they considered these elements less important compared to arthritis-related pain score and PtGA. This indicates that although providers were collecting some data elements, not all elements were reliably collected.

Positive experiences and acceptability have been reported by the majority of patients/caregivers, especially when considering factors like the distance of patients' residence from the healthcare provider, patients' educational level and the perceived benefits for social distancing (39, 40). In addition to reduced travel time, decreased missed time from work/school and financial savings associated with in-person visits, patients reported ease of use, shorter waiting periods and possible continued use in the post- pandemic period (26, 27, 41–43). Healthcare providers also reported high satisfaction, especially when patients had reliable internet (44). Common barriers identified with practicing telemedicine include lack of physical examination, reduced diagnostic accuracy due to incomplete clinical information, difficulty reaching patients, missing nonverbal communication, and lack of or challenges using technology required for telemedicine visits (45, 46). Barriers unique to the pediatric rheumatology population include trying to keep very young patients focused during virtual physical exam, and difficulty assessing psychosocial factors in adolescents when caregivers are present (46–48). Unfortunately, lower socioeconomic status and lower educational background may affect access to and quality of telemedicine visits e.g., poor bandwidth, which has implications in continuity of care, medication adherence and disease control (49–51). The quality of virtual care may also depend on the specific disease and its activity level. A randomized controlled trial demonstrated that telemedicine visits were not inferior to in-person visits for adult patients with rheumatoid arthritis whose disease was in remission or had low disease activity (52).

Although barriers to data collection were in part due to the nature of telemedicine and limitations in exam, a large contributor was simply due to provider workflow issues. The inexperience and lack of training in using telemedicine platforms, completing virtual patient check-ins, performing physical exams in a virtual setting, and the lack of support collecting patient reported outcomes, impacted their ability to collect CDE and complete their documentation.

Further, survey results indicated that for specific elements there were two main barriers: (1) the inability to conduct a reliable joint assessment that includes direct palpation of joints (especially when patients were not present at the visit), and (2) providers forgetting to collect and document the pertinent data elements. This illustrates that although telemedicine has limitations for specific aspects to the musculoskeletal exam, there are opportunities to improve workflows to collect the non-exam dependent, patient-reported data elements such as the PtGA or pain scores. As providers continue to integrate telemedicine as part of their clinical practice, we will need to consider systematic approaches to address these barriers, such as allocating job responsibilities and establishing force functions to ensure the reliable collection of CDE.

As previously indicated, fewer providers were comfortable performing physical examination to ascertain active joint count during telemedicine visit compared to acquiring other CDE due to the possibility of limited accuracy of the results. To address this concern, some providers may consider triaging patients to determine whether they should be seen virtually over telemedicine or if they should be seen in-person. To our knowledge, there is no universal established criteria on how to triage patients for telemedicine visits. One PR-COIN site utilized a pre-COVID developed triage tool that was developed prior to the pandemic which triaged based on referring symptoms to determine the urgency, time to be seen with the highest triaged levels 1 and 2 requiring in-person visit (53). Further research is also needed to identify which patients are most suitable to be seen for virtual visits and which might be better served by in-person assessment.

Alternatively, we may consider additional tools, models of care, and/or caregiver-specific education to facilitate the reliable reporting of physical examination results, including the active joint count. For example, there are already recommended modifications to the p-GALS, known as Virtual or Video-pGALS (V-pGALS), incorporating amended or additional maneuvers added to capture needed elements more accurately (47, 54). A pilot study has demonstrated the acceptability and reliability of this tool (31). Additional research needs to be performed to further validate the ability of the V-pGALS to perform joint assessment. This could be accomplished by performing a study where patients received a joint count over telemedicine followed by an in-person assessment shortly thereafter.

There is an opportunity to improve the collection of CDE that are not dependent on the clinical exam, such as patient-reported outcomes, over telemedicine. The introduction of new clinical workflows such as the incorporation of integrated electronic health record tools (for both providers and patients navigators), provider education with time sensitive scripting and checklists, medical staff virtual rooming protocols for medical staff, and pre-visit planning, may better support reliable collection of these metrics rather than forgetting. Enabling patients and proxies to take a proactive role in their healthcare by educating them on how to support their telemedicine visit and teaching them skill may empower them whilst improving the overall outcome of the telemedicine visit.

Despite being one of the ranked one of the most difficult CDE to collect via telemedicine, respondents indicated that active joint count was the most important CDE to collect over telemedicine. Given this opinion, additional efforts should be expended to improve the ability to accurately collect this variable. Recognizing that the varying levels of knowledge and technology literacy, educational curriculums should be carefully designed to ensure that healthcare providers possess the necessary knowledge and skillset to effectively provide care over telemedicine. Furthermore, the development of additional educational electronic tools i.e., phone applications, could improve timely access to providers.

It would be worth surveying patients to understand their opinion of healthcare delivery over telemedicine and their satisfaction with the process. Some studies have indicated that although being seen over telemedicine was preferred during the pandemic, it is not preferred after the pandemic (41, 42). Additional patient reported outcome measures/surveys could be introduced through patient portal builds in the electronic medical record.

Differentiating data that is collected by telemedicine to that from in-person visits will enable the comparison of patient outcomes to determine whether the delivery of care using telemedicine results in similar patient outcomes. This information will inform whether providing care over telemedicine is comparable to that in-person care or it may identify situations where telemedicine care is a satisfactory option.

Our study is limited by the fact that it surveyed the PR-COIN learning network. PR-COIN sites have previously collected CDE during in-person visits and they have already engrained this practice into their established workflows, practices, and culture. Therefore, these findings may be biased due to the active recall design of the survey, as well as the heightened awareness and prior collection of these data elements for clinical care. As such, these findings may not be representative of the broader pediatric rheumatology community. Broader surveys and studies involving the use of these data elements, both in in-person and virtual settings, amongst pediatric rheumatologists are required.

In addition, respondents answered questions based on their own practice. We did not inquire about the composition of their practice, such as the proportion of JIA subtypes seen in their clinic or the age range of their patient population. These factors may have influenced their responses. If their practice consisted primarily of adolescents with arthritis affecting larger joints, it may be easier to perform a virtual assessments may have been easier since because they can follow instructions, and the joint swelling would be more prominent, in contrast to a toddler with arthritis affecting small joints who is unable to follow instructions.

Although the majority of video platforms used in telemedicine have matured over time, they may vary in terms of available features and ease of use. These differences can influence the technical system requirements needed to operate the software or the user's learning curve.

It is also possible that self-reported collection of data elements may not accurately reflect actual practices, potentially over or underestimating actual practices. Collecting objective data on the frequency that these metrics are captured during visits would more definitively identify gaps. Additionally, while this survey primarily captures largely the provider experience with collecting data elements via telemedicine, future next steps may want to examine patient acceptability regarding the ways in which patient-reported outcomes are collected and utilized in telemedicine care. Ultimately, a deeper understanding of how collection of these data elements are collected and utilized, and how they affect patient clinical outcomes in JIA is needed and is currently being investigated currently underway.

It is important to remember that although it may be easy for some healthcare institutions to offer telemedicine to patients, health inequities still exist. These disparities can affect some individuals' ability to access care using this medium (21, 55). Additional steps must be taken to ensure equitable healthcare delivery using telemedicine in the future (25, 29, 56).

## Conclusion

Multiple barriers and facilitators exist in the delivery of pediatric rheumatology care over telemedicine. Our findings suggest that telemedicine processes and practices vary both across different centers, as well as within individual centers. This highlights the need to standardize telemedicine visit procedures to ensure that CDE are reliably and consistently collected, irrespective of visit type. Given that a portion of patients with JIA will likely continue to be serviced over telemedicine post-pandemic, teams need to adapt and refine their existing clinical practices to continue providing high-quality care using this platform.

## Data Availability

The raw data supporting the conclusions of this article will be made available by the authors, without undue reservation.
